# Real-World Health Monitoring Using Wrist-Worn Wearables in Community-Dwelling Older Adults in China: Mixed Methods Feasibility Study

**DOI:** 10.2196/98592

**Published:** 2026-10-29

**Authors:** Ying Bai, Yi Tang, Yiwei Zhao, Lixin Ma, Yanjun Ma, Zhibin Wang

**Affiliations:** 1 Department of Neurology & Innovation Center for Neurological Disorders, Xuanwu Hospital, Capital Medical University, National Center for Neurological Disorders, 45 Changchun Street, Xicheng District, Beijing, 100053, China, +86 13811021432; 2 Beijing Key Laboratory of Digital Medicine for Cognitive Disorders, Beijing, China; 3 Department of Neurology, The First Hospital of Hebei Medical University, Shijiazhuang, Hebei, China; 4 Xuanwu Hospital, Capital Medical University, National Center for Neurological Disorders, Beijing, China

**Keywords:** wearable devices, older adults, feasibility, usability, acceptability, digital health, China

## Abstract

**Background:**

Real-world behavioral and physiological functioning, including heart rate, daily mobility, and sleep patterns, may provide information about aging and early neurodegenerative changes that is not captured by periodic clinical visits. Wrist-worn wearable devices offer a promising approach for continuous, passive monitoring of these domains. However, evidence regarding feasibility, usability, and acceptability in real-world observational settings among community-dwelling older adults in China remains limited.

**Objective:**

This study primarily aimed to evaluate the feasibility, usability, and acceptability of using wrist-worn wearable devices to monitor behavioral and physiological function in community-dwelling older adults under naturalistic conditions. As a secondary objective, the study descriptively characterized wearable-derived behavioral and physiological measures among participants with minimal consecutive valid monitoring.

**Methods:**

We enrolled 486 community-dwelling older adults from the Beijing region of the China-Aging cohort, each of whom received a Huawei Band 8 commercial wrist-worn wearable device. A valid monitoring day was defined as 10 or more hours of wear time. Participants were classified into three monitoring-pattern categories based on predefined wear-time criteria: no valid monitoring day, nonconsecutive valid monitoring, and minimal consecutive valid monitoring (≥3 consecutive valid monitoring days). Demographic characteristics, health status, and cognitive and functional measures (Mini-Mental State Examination and Barthel Activities of Daily Living Index) were compared across groups. Usability, acceptability, and perceived barriers were assessed using a structured user experience survey.

**Results:**

Of 486 participants, 224 (46.1%) achieved minimal consecutive valid monitoring, 156 (32.1%) had nonconsecutive valid monitoring, and 106 (21.8%) had no valid monitoring day. Self-rated health differed significantly across monitoring patterns (*P*=.005), whereas no significant differences were observed for the other measured baseline characteristics. Among participants with minimal consecutive valid monitoring, mean daily heart rate was 72.1 (SD 8.6) bpm, median daily step count was 6177 (IQR 4249‐8872) steps/day, and sleep data were available for 211 out of 224 (94.2%), with a median sleep duration of 7.23 (IQR 6.48‐8.04) hours. The user experience survey was completed by 106 out of 486 (21.8%) participants, with higher participation among those with more sustained monitoring. Respondents generally reported favorable usability and acceptability; common barriers included device discomfort, data synchronization issues, and battery life.

**Conclusions:**

Wrist-worn wearable monitoring was feasible under naturalistic conditions for a substantial proportion of community-dwelling older adults in China, with generally favorable usability and acceptability among survey respondents. However, monitoring patterns varied considerably across individuals, highlighting challenges for long-term use and large-scale implementation. These findings support the potential of wearable technologies for longitudinal monitoring outside clinic-based assessments. Further research is needed to establish the stability and clinical relevance of wearable-derived behavioral measures over longer monitoring periods.

## Introduction

Population aging is a major global demographic trend that is reshaping public health priorities and increasing demands on health care systems worldwide [1]. As life expectancy increases, a growing proportion of older adults experience age-related declines in functional ability, reflecting gradual changes in physical and cognitive capacities that are essential for maintaining independent living [2]. Functional decline is strongly associated with increased morbidity, mortality, health care utilization, and societal burden [3,4].

Functional deterioration in later life rarely occurs abruptly. Accumulating evidence indicates that subtle and progressive changes in daily-life behaviors, such as alterations in walking patterns [5], physical activity levels [6], and sleep characteristics [7,8], often precede clinically detectable impairment by several years. Longitudinal and meta-analysis studies further suggest that increased variability in routine behaviors, particularly sleep and activity patterns, is associated with emerging cognitive and functional vulnerability, even in the absence of overt clinical symptoms [8-10].

Despite growing recognition of early functional change, current assessment approaches remain largely limited to episodic clinic-based assessments and self-reported measures [11]. Standard assessments typically rely on brief performance tests administered during scheduled clinical visits, retrospective questionnaires, or caregiver reports, providing only snapshots of functioning at isolated time points [11,12]. As a result, these approaches are limited in their ability to capture gradual trajectories of functional change, particularly during preclinical or early stages when deviations from individual baselines are subtle, heterogeneous, and context-dependent [12,13]. Moreover, self-reported and proxy-reported measures are vulnerable to recall bias and reporting inaccuracies, constraining sensitivity to small but clinically meaningful behavioral alterations [14,15].

Wearable sensing technologies have emerged as a promising approach to addressing these limitations. Wrist-worn devices can passively and continuously capture daily behaviors, including physical activity [16], sleep [17], and heart rate dynamics [18], using embedded accelerometers and photoplethysmography (PPG) sensors with minimal user burden [16,19]. Beyond passive data collection, accumulating evidence suggests that these continuously monitored behaviors carry prognostic significance [5,7,10].

Wearable-derived behavioral and physiological patterns show strong clinical associations in older adults. Lower daily step counts, reduced activity, and more sedentary profiles are associated with higher all-cause mortality and cardiovascular risk [4,20-22]. Similarly, sleep characteristics, including shorter duration, greater night-to-night variability, and longitudinal deterioration, are associated with subsequent cognitive impairment and neurodegenerative risk [7,8,23]. Recent work suggests that real-world behavioral measures derived from wrist-worn devices, including walking speed, activity patterns, and sleep timing, can predict incident dementia years before clinical diagnosis [5]. In particular, gait-related measures have been developed as digital biomarkers [24] and can distinguish mild Alzheimer disease from cognitively normal controls in community settings [25]. Beyond observational associations, wearable technologies are increasingly being applied in intervention studies and structured remote monitoring programs in clinical research [26-29]. Together, these findings support the potential value of wearable-derived measures for longitudinal health monitoring in older adults. However, their use depends in part on whether adequate monitoring can be achieved under real-world conditions [30,31].

However, important gaps remain in understanding real-world feasibility among older adults. Most feasibility studies have been conducted over short durations or under controlled conditions, often involving intensive researcher support, structured onboarding, frequent follow-up, or incentives [16,32]. Study samples are also frequently highly selected, excluding individuals with lower digital literacy, comorbidities, or functional limitations, thereby limiting generalizability [30,33]. While such approaches are appropriate for early-stage validation, they may not reflect feasibility under naturalistic, everyday conditions, where ongoing researcher support may be limited and users vary in their capabilities and support needs [32,34]. Additionally, feasibility data from Chinese community settings remain particularly limited, despite China’s rapidly aging population and increasing policy interest in digital health monitoring for older adults.

Accordingly, this study aimed to evaluate the feasibility, usability, and acceptability of wearable monitoring among community-dwelling older adults under naturalistic conditions by examining (1) the initiation and maintenance of wearable monitoring, (2) the proportion of participants achieving the predefined minimum criterion for consecutive valid monitoring, (3) measured participant characteristics associated with different wearable monitoring patterns, and (4) usability experiences and perceived barriers reported by participants who completed the user experience survey.

## Methods

### Study Design

This study was conducted within the China Healthy Aging Cohort Study (China-Aging; ClinicalTrials.gov identifier: NCT06472674), an ongoing longitudinal, multicenter cohort of community-dwelling older adults across several regions in China [35]. The China-Aging study aims to investigate the prevalence and risk factors of disability and cognitive impairment and to develop predictive models by integrating traditional clinical measures with technology-derived data.

The wearable monitoring component was established within the Beijing center of the China-Aging cohort to collect longitudinal, real-world behavioral and physiological data using wrist-worn wearable devices. The present study reports the feasibility, usability, and acceptability of this monitoring approach under naturalistic conditions. A mixed methods design was used, combining quantitative wearable monitoring data with user experience survey data, including open-ended responses analyzed thematically. Baseline demographic and clinical assessments were conducted by trained researchers as part of the China-Aging cohort procedures.

### Ethical Considerations

The study was approved by the Ethics Committee of Xuanwu Hospital, Capital Medical University (approval number 2024214001), and all participants provided written informed consent prior to participation.

### Participants

Participants were recruited from the Beijing region of the China-Aging cohort through the Baizhifang Community Health Centre. Eligible participants were invited to take part in this wearable monitoring study after completing baseline assessments within the cohort.

Inclusion criteria were (1) age 55 years or above, (2) residence in the local community for at least 1 year, (3) ability to independently use a wrist-worn device, (4) ownership of a compatible Android smartphone for successful device pairing, and (5) ability to install and use the Huawei Health app with regular access to Wi-Fi or mobile data for data synchronization. Exclusion criteria included (1) unstable medical conditions or acute illness at the time of recruitment, (2) severe cervical or lumbar spine disease or advanced osteoarthritis substantially limiting routine daily activity, and (3) incomplete baseline assessment.

Baseline assessments included demographic characteristics (age, sex, education level, marital status, living arrangement, care arrangement, and self-rated health status), cognitive function assessed using the Mini-Mental State Examination (MMSE) [36], and functional status assessed using the Barthel Activities of Daily Living (ADL) Index [37]. All enrolled participants demonstrated sufficient cognitive and functional capacity for independent device use at baseline.

### Study Procedure

Wearable devices were distributed beginning in December 2023, with rolling enrollment following the completion of baseline assessments. Devices were offered consecutively to eligible and willing participants after the confirmation of smartphone compatibility and successful completion of device pairing, connection testing, and data transmission. Participants were not selected based on demographic characteristics, self-rated health status, or prior technology experience.

Participants received the wearable device at no cost and were instructed to wear it during daily life without restrictions, including during sleep, removing it only when necessary (eg, for charging). The monitoring start date was defined as the day on which participants completed device activation and first wore the device following study onboarding procedures. No predefined monitoring duration or mandatory wear requirements were set, and participants were allowed to discontinue device use at any time without affecting their participation in the China-Aging cohort. Monitoring continued until the last successful data synchronization or the end of November 2025, whichever occurred first.

In-person technical support was provided during initial device setup, with subsequent troubleshooting available through a WeChat group. Brief reminders were sent approximately every 2 days during the first 2 weeks to support device activation and data synchronization and to identify early technical issues. Thereafter, no proactive reminders or adherence-enhancing contact were provided, although participant-initiated technical support remained available through WeChat. This limited-support approach was intended to approximate real-world wearable use under naturalistic conditions.

### Wearable Device and Data Acquisition

Participants were provided with a commercial wrist-worn wearable device (Huawei Band 8; Huawei Technologies Co) designed for the continuous monitoring of heart rate, physical activity, and sleep. The device incorporates a PPG sensor for heart rate monitoring and a triaxial accelerometer for activity tracking. Proprietary onboard algorithms generate summary measures of heart rate, step counts, and sleep parameters as implemented by the device manufacturer.

Participants paired the device with the Huawei Health app on their personal smartphones, through which wearable data were transmitted via Bluetooth and synchronized to the Huawei Health Research Platform. Data available through the research platform were pseudonymized and identified by wearable device ID. The linkage between wearable device ID and participant number was maintained separately by the research team. Data exported for analysis were stored on a secure research laptop accessible only to the study team.

### Wear-Time Definition and Monitoring Criteria

Minute-level heart rate data were used as the primary indicator of wear-time, as heart rate is recorded only during direct skin contact [17,38]. Brief data gaps may occur due to transient loss of sensor contact or motion-related artifacts. Consistent with the improved nonwear detection algorithm proposed by Choi et al [39], which extended the foundational approach of Troiano et al [40], short interruptions of 1‐2 minutes were treated as continuous wear, whereas longer gaps were considered potential nonwear periods. In the present study, nonwear was defined as intervals of 90 or above consecutive minutes without valid heart rate data, and subsequent data were treated as a new wear period. This approach was further reviewed and confirmed through consultation with Huawei technical engineers. Accordingly, daily wear-time was calculated as the sum of all valid wear periods within each calendar day.

A valid monitoring day was defined as a day with 10 or more hours of wear time, consistent with definitions used in wearable and accelerometry research [33,41]. A minimum of 3 valid monitoring days has been used in previous wearable research [33]. In the present feasibility study, we additionally required these 3 valid days to be consecutive as a pragmatic operational criterion for identifying short-term monitoring continuity under naturalistic conditions.

Based on these criteria, participants were classified into three monitoring-pattern categories:

1. No valid monitoring day: no day met the ≥10-hour valid-day criterion.
2. Nonconsecutive valid monitoring: 1 or more valid monitoring days, but no sequence of 3 or more consecutive valid monitoring days.
3. Minimal consecutive valid monitoring: 3 or more consecutive valid monitoring days.

### User Experience Survey

User acceptability and usability were assessed using a self-administered questionnaire following a period of real-world wearable use. The questionnaire was developed for this study, informed by existing wearable usability instruments [32,42], and designed to capture multiple dimensions of usability, acceptability, and perceived impact in real-world use. It comprised 16 items, including 13 Likert-scale questions covering device comfort, ease of use, display readability, charging, app connectivity, data recording and accuracy, perceived lifestyle impact, and willingness for continued use and data sharing, as well as 3 open-ended questions exploring perceived benefits, barriers, and suggestions for improvement.

All Likert-scale items were rated on a 5-point scale, with higher scores indicating more positive evaluations. The questionnaire was originally developed in Chinese and translated into English for reporting. It was distributed to all participants after an initial period of device use, and responses were collected for subsequent analysis. Full details of the questionnaire are provided in Multimedia Appendix 1.

### Statistical Analysis

All analyses were conducted using R (version 4.5.1) [43]. Descriptive statistics were used to summarize participant characteristics, monitoring metrics (eg, wear-time and valid days), and wearable-derived measures (eg, heart rate, physical activity, and sleep). Continuous variables are reported as mean (SD) for normally distributed data and median (IQR) for nonnormally distributed data.

Group comparisons were performed to examine potential differences in demographic, self-rated health status; cognitive function; and functional characteristics associated with wearable monitoring patterns. The distributions of continuous variables were assessed using the Shapiro-Wilk test. Continuous variables were summarized using median (IQR) and compared across monitoring-pattern groups using the Kruskal-Wallis test, with epsilon-squared (*ε*²) reported as the effect size. Categorical variables were compared using Pearson chi-square tests or exact tests where appropriate, with Cramér *V* reported as the effect size. These analyses were exploratory and intended to characterize potential differences associated with monitoring patterns. Statistical significance was defined as a 2-sided *P*<.05.

For survey data, responses to Likert-scale items were summarized descriptively using median (IQR) and mean (SD). Open-ended responses were analyzed using an inductive thematic approach. Two researchers (YB and YZ) independently reviewed the responses and developed initial thematic categories related to perceived benefits, barriers, and suggested improvements. They then compared their categorizations and discussed disagreements, refining the categories until consensus was reached on the final coding and thematic structure.

To assess the robustness and potential sources of selection bias, we examined alternative monitoring thresholds and continuous monitoring measures, compared baseline characteristics between enrolled and nonenrolled participants, and compared baseline characteristics and monitoring measures between survey respondents and nonrespondents. Details of these analyses are provided in Multimedia Appendix 2.

## Results

### Participant Characteristics

All 710 participants approached for the wearable study owned a smartphone. Of these, 147 used a phone that was incompatible with the Huawei Health app (eg, iOS devices or unsupported Android versions), 64 did not proceed because stable data transmission could not be established during connection testing, and 13 did not enroll for other reasons that were not systematically recorded. Refusal was not recorded as a separate category and may therefore have been included among these 13 participants. Ultimately, 486 participants received a Huawei Band 8 and entered the wearable study, with wearable data available for all enrolled participants.

Baseline comparisons between the 486 enrolled and 224 nonenrolled participants showed no significant differences in age, sex, education, marital status, or self-rated health. Enrolled participants had higher MMSE scores and differed in living arrangement and care arrangement; a statistically significant but small difference was also observed in ADL. Full results are provided in Multimedia Appendix 2.

Among the 486 enrolled participants, 224 (46.1%) achieved minimal consecutive valid monitoring (≥3 consecutive valid monitoring days), 156 (32.1%) had nonconsecutive valid monitoring, and 106 (21.8%) had no valid monitoring day. Among participants with no valid monitoring day, 97 of 106 (91.5%) participants had a maximum observed daily wear time of less than 1 hour, while the remaining 9 of 106 (8.5%) participants had a maximum daily wear time of 1 to less than 5 hours. Within the nonconsecutive valid monitoring group, 119 of 156 (76.3%) participants had 1 valid monitoring day, 29 of 156 (18.6%) participants had 2 valid monitoring days, and 8 of 156 (5.1%) participants accumulated 3 or more valid monitoring days but never achieved 3 consecutively. Overall, 148 of 156 (94.9%) participants in this group had only 1 or 2 valid monitoring days. Full results are provided in Multimedia Appendix 2.

Baseline demographic, self-rated health status, cognitive, and functional characteristics across the 3 monitoring patterns are summarized in Table 1. The observed age range among enrolled participants was 59 to 86 years. Age, sex, education level, marital status, living arrangement, care arrangement, MMSE score, and Barthel ADL score did not differ significantly across monitoring patterns (all *P*>.05). Self-rated health was the only measured baseline characteristic that differed significantly across groups (Cramér *V*=0.114; *P*=.005), with better self-rated health more frequently reported among participants with minimal consecutive valid monitoring.

**Table 1.** Baseline characteristics by wearable monitoring pattern (N=486). Effect sizes are reported as *ε*^2^ for Kruskal-Wallis tests, and Cramér V for chi-square tests.

Characteristic	No valid monitoring day (n=106)	Nonconsecutive valid monitoring (n=156)	Minimal consecutive valid monitoring (n=224)	*P* value	Effect size
Age (years), median (IQR; range)	69 (66‐72; 60‐83)	68 (65‐73; 60‐86)	68 (65‐71; 59‐85)	.07	0.007^a^
Female sex, n (%)	65 (61.3)	94 (60.3)	155 (69.2)	.15^b^	0.062^c^
Education level, n (%)				.30^b^	0.040^c^
At or below junior high school	35 (33)	50 (32.1)	57 (25.4)		
High school/technical secondary	36 (34)	60 (38.5)	79 (35.3)		
At or above college/undergraduate	35 (33)	46 (29.5)	88 (39.3)		
Marital status, n (%)				.15^b^^,d^	0.078^c^
Married	98 (92.5)	145 (92.9)	213 (95.1)		
Not currently married^e^	8 (7.5)	11 (7.1)	11 (4.9)		
Living arrangement, n (%)				.97^b^	0.000^c^
With spouse only	76 (71.7)	107 (68.6)	151 (67.4)		
With children only	9 (8.5)	18 (11.5)	24 (10.7)		
With spouse and children	7 (6.6)	12 (7.7)	18 (8)		
With relatives/friends	12 (11.3)	15 (9.6)	28 (12.5)		
Living alone	2 (1.9)	4 (2.6)	3 (1.3)		
Care arrangement, n (%)				.97^b^	0.000^c^
No caregiver	72 (67.9)	99 (63.5)	148 (66.1)		
Spouse caregiver	32 (30.2)	51 (32.7)	67 (29.9)		
Child caregiver	2 (1.9)	6 (3.8)	7 (3.1)		
Relative/friend caregiver	—^f^	—	1 (0.4)		
Other	—	—	1 (0.4)		
Self-rated health status, n (%)				.005^b^	0.114^c^
Good or very good	18 (17)	36 (23.1)	71 (31.7)		
Fair	75 (70.8)	105 (67.3)	143 (63.8)		
Poor or very poor	13 (12.3)	15 (9.6)	10 (4.5)		
MMSE^g^ score, median (IQR)	29 (27‐29)	29 (27‐29)	29 (27‐30)	.09^h^	0.006^a^
Barthel ADL^i^ Index, median (IQR)	100 (95‐100)	100 (95‐100)	100 (95‐100)	.95^h^	0.000^a^

a Epsilon-squared.

b Chi-square test.

c Cramér V.

d Interpret with caution due to small cell counts.

e Includes single, divorced, and widowed.

f Not applicable.

g MMSE: Mini-Mental State Examination.

h Kruskal-Wallis test.

i ADL: Activities of Daily Living.

### Wearable Monitoring Patterns and Data Availability

Among the 224 participants with minimal consecutive valid monitoring, the median total number of valid monitoring days was 60.5 (IQR 19‐94.3; range 3‐409), while the median maximum consecutive valid-day streak was 16 days (IQR 8‐34; range 3‐168), indicating substantial heterogeneity in both monitoring extent and continuity (Figure 1).

**Figure 1.**
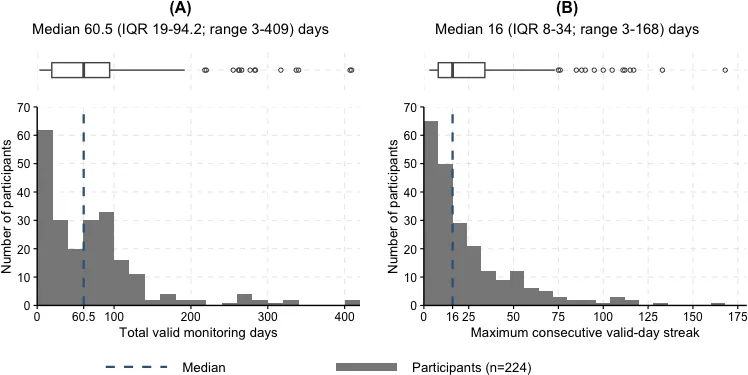
Monitoring extent and continuity among participants with minimal consecutive valid monitoring. (A) Distribution of total valid monitoring days. (B) Distribution of the maximum consecutive valid-day streak. Histograms show the number of participants across monitoring durations, with dashed blue lines indicating the median. Boxplots show the median, IQR, and distributional spread; circles indicate outliers.

Sensitivity analyses were conducted to examine the robustness of the findings to the primary ≥3-consecutive-valid-day criterion. Among the 380 participants with 1 or more valid monitoring days, 224 (58.9%), 211 (55.5%), 196 (51.6%), and 178 (46.8%) achieved 3 or more, 4 or more, 5 or more, and 7 or more consecutive valid monitoring days, respectively. The association between better self-rated health and consecutive valid monitoring remained directionally consistent across these alternative thresholds. Full results of the sensitivity analyses are provided in Multimedia Appendix 2.

Among participants with minimal consecutive valid monitoring, heart rate and step-count data were available for all 224 participants. Mean daily heart rate was 72.1 (SD 8.6) bpm, and median daily step count was 6177 (IQR 4249‐8872) steps/day. Sleep data were available for 211 of 224 (94.2%) participants, with a median sleep duration of 7.23 (IQR 6.48‐8.04) hours (Table 2).

**Table 2.** Summary of wearable-derived signal availability and characteristics among participants with minimal consecutive valid monitoring.

Signal	Participants with available data, n (%)	Summary metrics
Heart rate	224 (100)	72.1 (8.6; 49.4‐93.1)^a^ bpm
Sleep	211 (94.2)	7.23 (6.48‐8.04; 3.22‐10.39)^b^ hours
Step count	224 (100)	6177 (4249‐8872; 269‐19,067)^b^ steps/day

a Mean daily heart rate (SD; range).

b Median (IQR; range).

### User Experience Survey

User acceptability and experience were assessed using a self-administered questionnaire completed by 106 of 486 (21.8%) participants after a period of real-world device use. Survey response rates were higher among participants with minimal consecutive valid monitoring (74/224, 33%) than among those with nonconsecutive valid monitoring (21/156, 13.5%) or no valid monitoring days (11/106, 10.4%). Although respondents and nonrespondents were broadly comparable in measured baseline characteristics (Multimedia Appendix 2), respondents demonstrated greater monitoring continuity and persistence. The survey findings therefore primarily reflect the experiences of participants who maintained greater wearable use.

Across the 13 Likert-scale items (n=106), ratings were generally above the neutral midpoint (score=3), indicating overall favorable usability and acceptability (see Multimedia Appendix 3 for full item-level results). Median scores were 4 for most items, with IQRs ranging from 3-4 to 4-5, and mean values ranging from 3.51 to 4.22, with SDs ranging from 0.59 to 1.09. The highest rating was observed for independence of use (Q6; mean 4.22, SD 0.96), suggesting that most participants were able to operate the device without assistance. Items related to usability and data perception, including comfort, ease of use, readability, charging, connectivity, accuracy, and trust (Q1-Q8), also showed consistently positive ratings.

Perceived impact on health behavior was more varied. While participants reported increased health awareness (Q9) and greater willingness to engage in physical activity (Q10), perceived changes in daily habits (Q11) were more modest (median 3), indicating greater variability in responses. This suggests that although respondents reported greater awareness and motivation, perceived changes in daily habits were more limited. Willingness for long-term use (Q12) and data sharing with health care providers (Q13) remained high (median 4, IQR 4-4), indicating generally favorable acceptability among respondents.

For the open-ended questions, participants reported both perceived benefits and barriers to device use. The most commonly reported benefits included improved sleep monitoring (38/106, 35.8%) and activity or exercise tracking (37/106, 34.9%), followed by increased awareness of heart rate and oxygen saturation (29/106, 27.4%) and greater attention to overall health management (29/106, 27.4%). Only a small proportion (4/106, 3.8%) of participants reported no perceived benefits.

Reported barriers included device discomfort (18/106, 17%), data synchronization issues (12/106, 11.3%), and limited battery life (12/106, 11.3%). Some participants noted that removing the device for charging or showering disrupted routines and increased the likelihood of forgetting to put it back on. Others reported difficulty using functions beyond basic activity tracking. In contrast, 36 of 106 (34%) participants reported no inconvenience and were satisfied with device use.

Suggestions for improvement focused on both functionality and usability. The most common suggestions included adding additional health monitoring features (35/106, 33%), such as blood pressure or glucose tracking, simplifying device operation and guidance (17/106, 16%), and improving device comfort and battery performance (12/106, 11.3%). Approximately one quarter (27/106, 25.5%) of respondents reported no additional suggestions and were satisfied with the current device. A detailed breakdown of thematic categories is provided in Multimedia Appendix 3.

## Discussion

### Principal Findings

This study evaluated the real-world feasibility, usability, and acceptability of using a consumer wearable device for continuous health monitoring in community-dwelling older adults under limited-support, naturalistic conditions. The findings indicate that wearable monitoring was feasible for a substantial proportion of participants, with many achieving consecutive valid monitoring and maintaining device use beyond the initial monitoring period. At the same time, monitoring patterns varied considerably across individuals, with substantial heterogeneity in both monitoring extent and continuity, highlighting challenges for sustained real-world monitoring and large-scale implementation.

The observed monitoring patterns should be interpreted in the context of the limited-support design. Participants received support during initial device setup and brief reminders during the first 2 weeks but no proactive adherence support thereafter. This differs from previous studies using structured follow-up, training, prompting, or incentives and reporting relatively high adherence [16,32,44,45]. For example, Paolillo et al [45] reported wearable use on an average of 95% of study days during a supported 30-day protocol, while Holmqvist et al [44] reported a mean daily wear time of 21 hours during a structured 4-week study involving training and automated prompting. Although direct comparisons are limited by differences in study design and monitoring definitions, the present findings provide an estimate of wearable use under conditions with limited ongoing support. The substantial variation in monitoring extent and continuity also aligns with broader patterns of digital health use, in which sustained engagement depends on the integration of technology into everyday routines [46,47]. Together, these findings suggest that wearable feasibility is influenced by implementation context and support intensity and that supported and naturalistic studies provide complementary evidence for understanding real-world use.

### Implementation Challenges and Associated Factors

From an implementation perspective, participants with nonconsecutive valid monitoring may represent an important target for improving sustained wearable use. Most participants in this group accumulated only 1 or 2 valid monitoring days, suggesting that initial device use did not consistently develop into sustained monitoring [48,49]. Practical barriers identified in the user experience survey, including data synchronization, battery management, and interruptions associated with device removal, may contribute to difficulties in incorporating wearable use into everyday routines [29,32,50]. Previous research suggests that sustained wearable use can be supported by the development of routine behaviors, such as integrating device wear and charging into established daily activities [50,51]. Additional support during the early monitoring period may therefore be one strategy for facilitating the transition from initial to more sustained use.

Participants with no valid monitoring day may represent a different implementation challenge. Although all participants in this group generated some wearable data, observed wear was extremely limited: among the 106 participants, 97 (91.5%) participants had a maximum daily wear time of less than 1 hour, and none reached 5 hours on any observed day. This pattern suggests that the predominant challenge was establishing meaningful wearable use after initial setup, rather than wear that narrowly fell below the ≥10-hour valid-day criterion. Notably, this occurred despite successful device pairing, connection testing, and data transmission before enrollment, suggesting that successful technical onboarding alone may not be sufficient to ensure subsequent wearable use. Potential barriers may include technical and usability challenges, perceived usefulness, and difficulties integrating the device into everyday activities [29,32,52,53]. However, the specific reasons for limited monitoring in this group could not be determined because survey participation was particularly low, and barriers reported by survey respondents may not reflect the experiences of participants with little valid monitoring. Further research is therefore needed to identify the factors contributing to limited early-stage monitoring and determine whether targeted technical or usability support could facilitate continued device use.

Taken together, these findings suggest that different monitoring patterns may require different implementation strategies. Participants with some valid monitoring but limited continuity may benefit from support focused on establishing consistent monitoring routines, whereas those who do not achieve a valid monitoring day may require greater attention to barriers occurring earlier in the monitoring process. Distinguishing between these patterns may therefore help inform more targeted approaches to supporting wearable monitoring.

Self-rated health was the only measured baseline characteristic significantly associated with monitoring pattern, with better self-rated health more frequently reported among participants achieving minimal consecutive valid monitoring. This association remained directionally consistent across alternative consecutive-day thresholds, suggesting that the finding was not dependent on the primary ≥3-day criterion. Similar associations between health status and digital health use have been reported previously, with greater health burden potentially increasing the practical demands of device use [34,48,49]. Self-rated health is a multidimensional measure that may reflect aspects of physical and psychological well-being, perceived control, and health-related self-efficacy, which have also been associated with technology adoption and health behaviors [52,54]. However, the mechanisms underlying the observed association were not assessed in the present study, and the finding should therefore be interpreted as exploratory rather than causal.

The absence of significant associations between monitoring patterns and age, education, cognitive function, or functional status is also informative, suggesting that these measured characteristics did not substantially differentiate monitoring patterns within the enrolled cohort. However, these findings should be interpreted in the context of the relatively cognitively and functionally intact study population. The high and narrow range of MMSE and Barthel ADL scores limited the ability to examine the effects of greater cognitive or functional impairment. In addition, comparisons between enrolled and nonenrolled individuals identified differences in cognitive function, living arrangement, and care arrangement, indicating that selection may occur before wearable monitoring begins. Therefore, the absence of associations within the enrolled cohort should not be interpreted as evidence that cognitive or functional characteristics are not important for wearable adoption more broadly [52,53,55]. Further research including older adults with more diverse cognitive, functional, and digital capabilities is needed.

Among participants with minimal consecutive valid monitoring, wearable-derived heart rate and step-count data were available for all participants, while sleep data were available for 94.2% (n=211) of the participants. This indicates that multiple behavioral and physiological measures could be obtained among participants who achieved minimal consecutive valid monitoring. The slightly lower availability of sleep data may reflect the additional requirements of overnight wear, including charging and device removal. More broadly, these findings indicate that successful wearable monitoring does not necessarily result in equivalent data availability across monitoring domains, an important consideration when consumer wearables are used for longitudinal health monitoring.

The user experience survey also provided insight into practical aspects of device use. While some participants reported no inconvenience, others identified barriers related to comfort, data synchronization, and battery life, and 33% (35/106) of the respondents suggested additional health-monitoring functions, such as blood pressure or glucose monitoring. These findings suggest that both practical burden and perceived relevance may influence continued wearable use and that greater alignment between device functionality and users’ health priorities may enhance acceptability [29,32,46,47]. However, these findings should be interpreted with caution because the respondents demonstrated greater monitoring continuity and persistence than nonrespondents, indicating that the usability, acceptability, and barrier findings disproportionately reflect the experiences of participants who maintained greater wearable use.

### Strengths, Limitations, and Future Directions

The principal strength of this study lies in its community-based design under naturalistic conditions, which captured variability in wearable use with limited ongoing support. The combination of objective monitoring data and user experience responses also enabled feasibility to be examined from complementary perspectives. Several limitations should be considered. First, recruitment from an urban community setting and requirements for smartphone compatibility and successful data transmission may limit generalizability, particularly to older adults with lower digital capability or greater cognitive or functional impairment. Differences between enrolled and nonenrolled participants further indicate potential preenrollment selection. Second, the ≥3-consecutive-valid-day criterion was a pragmatic operational definition rather than a validated threshold, although sensitivity analyses using alternative thresholds and continuous monitoring measures supported the overall findings. Third, the relatively low user experience survey participation rate (106/486, 21.8%) and greater monitoring persistence among respondents may have resulted in more favorable estimates of usability and acceptability. In addition, individual-level use of technical support was not systematically recorded, limiting our ability to assess whether support use differed across monitoring patterns or influenced monitoring continuity. Finally, the study used a single consumer wearable ecosystem with proprietary algorithms that were not independently validated against research-grade reference measures in this population. Future research should therefore examine longer-term monitoring patterns, identify barriers to sustained use, evaluate targeted technical and behavioral support, and validate wearable-derived measures against reference standards. Longitudinal associations with cognitive, functional, and health outcomes should also be investigated to establish the potential clinical relevance of these measures.

### Conclusions

In conclusion, this study demonstrates that wrist-worn wearable monitoring is feasible for a substantial proportion of community-dwelling older adults in China under limited-support, naturalistic conditions, although monitoring extent and continuity varied considerably. Usability and acceptability were generally favorable among survey respondents. Future research should identify strategies to support sustained monitoring and establish the clinical relevance of wearable-derived measures for longitudinal changes in cognitive and functional health.

## Supplementary material

10.2196/98592 Multimedia Appendix 1 Wearable device usability and acceptability survey.

10.2196/98592 Multimedia Appendix 2 Summary of additional analyses on monitoring patterns, sensitivity analyses, and potential selection bias.

10.2196/98592 Multimedia Appendix 3 Summary of survey results for usability and acceptability.

## Abbreviations

ADL: Activities of Daily Living
MMSE: Mini-Mental State Examination
PPG: photoplethysmography

## References

[1] (2025). Ageing and health. World Health Organization.

[2] (2015). World report on ageing and health. https://www.who.int/publications/i/item/9789241565042.

[3] Newman AB (2023). The epidemiology and societal impact of aging-related functional limitations: a looming public health crisis. J Gerontol A Biol Sci Med Sci.

[4] Paluch AE, Bajpai S, Bassett DR (2022). Daily steps and all-cause mortality: a meta-analysis of 15 international cohorts. Lancet Public Health.

[5] Chan LLY, Espinoza Cerda MT, Brodie MA, Lord SR, Taylor ME (2025). Daily-life walking speed, running duration and bedtime from wrist-worn sensors predict incident dementia: a watch walk—UK biobank study. Int Psychogeriatr.

[6] Del Pozo Cruz B, Ahmadi M, Naismith SL, Stamatakis E (2022). Association of daily step count and intensity with incident dementia in 78 430 adults living in the UK. JAMA Neurol.

[7] Keil SA, Schindler AG, Wang MX (2023). Longitudinal sleep patterns and cognitive impairment in older adults. JAMA Netw Open.

[8] Xia Y, He S, Sun Z, Wang F (2026). The association between sleep variability and cognitive function: a systematic review and meta analysis. J Affect Disord.

[9] Chamberlin KW, Etzkorn LH, Davoudi A (2026). Interdaily variability of rest–activity patterns and risk of dementia in older adults: the ARIC Neurocognitive Study. Alzheimers Dement (N Y).

[10] Wang W, Wanigatunga AA, Etzkorn LH (2026). Association between circadian rest-activity rhythms and incident dementia in older adults: the atherosclerosis risk in communities study. Neurology.

[11] Piau A, Wild K, Mattek N, Kaye J (2019). Current state of digital biomarker technologies for real-life, home-based monitoring of cognitive function for mild cognitive impairment to mild Alzheimer disease and implications for clinical care: systematic review. J Med Internet Res.

[12] Shephard RJ (2003). Limits to the measurement of habitual physical activity by questionnaires. Br J Sports Med.

[13] González DA, Gonzales MM, Jennette KJ, Soble JR, Fongang B (2021). Cognitive screening with functional assessment improves diagnostic accuracy and attenuates bias. Alzheimers Dement (Amst).

[14] Jekel K, Damian M, Wattmo C (2015). Mild cognitive impairment and deficits in instrumental activities of daily living: a systematic review. Alzheimers Res Ther.

[15] Kroenke K, Stump TE, Monahan PO (2022). Agreement between older adult patient and caregiver proxy symptom reports. J Patient Rep Outcomes.

[16] Teixeira E, Fonseca H, Diniz-Sousa F (2021). Wearable devices for physical activity and healthcare monitoring in elderly people: a critical review. Geriatrics (Basel).

[17] de Zambotti M, Cellini N, Goldstone A, Colrain IM, Baker FC (2019). Wearable sleep technology in clinical and research settings. Med Sci Sports Exerc.

[18] Alugubelli N, Abuissa H, Roka A (2022). Wearable devices for remote monitoring of heart rate and heart rate variability—what we know and what is coming. Sensors (Basel).

[19] Lu L, Zhang J, Xie Y (2020). Wearable health devices in health care: narrative systematic review. JMIR Mhealth Uhealth.

[20] Ding D, Nguyen B, Nau T (2025). Daily steps and health outcomes in adults: a systematic review and dose-response meta-analysis. Lancet Public Health.

[21] Hamaya R, Shiroma Jr EJ, Moore CC, Buring JE, Evenson KR, Lee IM (2024). Time- vs step-based physical activity metrics for health. JAMA Intern Med.

[22] LaMonte MJ, LaCroix AZ, Nguyen S (2024). Accelerometer-measured physical activity, sedentary time, and heart failure risk in women aged 63 to 99 years. JAMA Cardiol.

[23] Winer JR, Mander BA, Helfrich RF (2019). Sleep as a potential biomarker of tau and β-amyloid burden in the human brain. J Neurosci.

[24] Chan LLY, Choi TCM, Lord SR, Brodie MA (2022). Development and large-scale validation of the Watch Walk wrist-worn digital gait biomarkers. Sci Rep.

[25] Varma VR, Ghosal R, Hillel I (2021). Continuous gait monitoring discriminates community-dwelling mild Alzheimer’s disease from cognitively normal controls. Alzheimers Dement (N Y).

[26] Kim HJ, Hwang YJ, Park JE, Lee DY (2025). Effectiveness of walking prescription using mobile health technology on the changes in daily steps in older adults with cognitive impairment: randomized controlled study. JMIR Aging.

[27] Lentzen M, Vairavan S, Muurling M (2025). RADAR-AD: assessment of multiple remote monitoring technologies for early detection of Alzheimer’s disease. Alzheimers Res Ther.

[28] Muurling M, de Boer C, Kozak R (2021). Remote monitoring technologies in Alzheimer’s disease: design of the RADAR-AD study. Alzheimers Res Ther.

[29] Muurling M, de Boer C, Hinds C (2024). Feasibility and usability of remote monitoring in Alzheimer’s disease. Digit Health.

[30] Mc Ardle R, Jabbar KA, Del Din S (2023). Using digital technology to quantify habitual physical activity in community dwellers with cognitive impairment: systematic review. J Med Internet Res.

[31] Ning Y, Chen M, Yang H, Jia J (2025). Accelerometer-measured physical activity timing with incident dementia. Alzheimers Dement.

[32] Keogh A, Dorn JF, Walsh L, Calvo F, Caulfield B (2020). Comparing the usability and acceptability of wearable sensors among older Irish adults in a real-world context: observational study. JMIR Mhealth Uhealth.

[33] Chan A, Chan D, Lee H, Ng CC, Yeo AHL (2022). Reporting adherence, validity and physical activity measures of wearable activity trackers in medical research: a systematic review. Int J Med Inform.

[34] Abouzahra M, Ghasemaghaei M (2020). The antecedents and results of seniors’ use of activity tracking wearable devices. Health Policy Technol.

[35] Chang J, Wu Y, Zhao Y (2025). Cohort profile: China healthy aging cohort study (China-Aging). J Alzheimers Dis.

[36] Folstein MF, Folstein SE, McHugh PR (1975). “Mini-mental state”. A practical method for grading the cognitive state of patients for the clinician. J Psychiatr Res.

[37] Mahoney FI, Barthel DW (1965). Functional evaluation: the Barthel Index. Md State Med J.

[38] Nelson BW, Low CA, Jacobson N, Areán P, Torous J, Allen NB (2020). Guidelines for wrist-worn consumer wearable assessment of heart rate in biobehavioral research. NPJ Digit Med.

[39] Choi L, Liu Z, Matthews CE, Buchowski MS (2011). Validation of accelerometer wear and nonwear time classification algorithm. Med Sci Sports Exerc.

[40] Troiano RP, Berrigan D, Dodd KW, Mâsse LC, Tilert T, McDowell M (2008). Physical activity in the United States measured by accelerometer. Med Sci Sports Exerc.

[41] Sasaki JE, Júnior JH, Meneguci J (2018). Number of days required for reliably estimating physical activity and sedentary behaviour from accelerometer data in older adults. J Sports Sci.

[42] Mercer K, Giangregorio L, Schneider E, Chilana P, Li M, Grindrod K (2016). Acceptance of commercially available wearable activity trackers among adults aged over 50 and with chronic illness: a mixed-methods evaluation. JMIR Mhealth Uhealth.

[43] The R Project for Statistical Computing.

[44] Holmqvist S, Kaplan M, Chaturvedi R, Shou H, Giovannetti T (2025). Longitudinal and combined smartwatch and ecological momentary assessment in racially diverse older adults: feasibility, adherence, and acceptability study. JMIR Hum Factors.

[45] Paolillo EW, Lee SY, VandeBunte A (2022). Wearable use in an observational study among older adults: adherence, feasibility, and effects of clinicodemographic factors. Front Digit Health.

[46] Eysenbach G (2005). The law of attrition. J Med Internet Res.

[47] Moore K, O’Shea E, Kenny L (2021). Older adults’ experiences with using wearable devices: qualitative systematic review and meta-synthesis. JMIR Mhealth Uhealth.

[48] Perski O, Blandford A, West R, Michie S (2017). Conceptualising engagement with digital behaviour change interventions: a systematic review using principles from critical interpretive synthesis. Transl Behav Med.

[49] Yardley L, Spring BJ, Riper H (2016). Understanding and promoting effective engagement with digital behavior change interventions. Am J Prev Med.

[50] Lally P, van Jaarsveld CHM, Potts HWW, Wardle J (2010). How are habits formed: modelling habit formation in the real world. Euro J Social Psych.

[51] Peng W, Li L, Kononova A, Cotten S, Kamp K, Bowen M (2021). Habit formation in wearable activity tracker use among older adults: qualitative study. JMIR Mhealth Uhealth.

[52] Czaja SJ, Charness N, Fisk AD (2006). Factors predicting the use of technology: findings from the Center for Research and Education on Aging and Technology Enhancement (CREATE). Psychol Aging.

[53] Vaportzis E, Clausen MG, Gow AJ (2017). Older adults perceptions of technology and barriers to interacting with tablet computers: a focus group study. Front Psychol.

[54] Heart T, Kalderon E (2013). Older adults: are they ready to adopt health-related ICT?. Int J Med Inform.

[55] Mitzner TL, Boron JB, Fausset CB (2010). Older adults talk technology: technology usage and attitudes. Comput Human Behav.

