# Supplementary material for: Real-World Health Monitoring Using Wrist-Worn Wearables in Community-Dwelling Older Adults in China: Mixed Methods Feasibility Study
Source: JMIR Hum Factors. 2026 Oct 29;13:e98592. doi: 10.2196/98592 (PMC13718885; doi:10.2196/98592)
Supplement: Supplementary file 1 [file humanfactors-v13-e98592-s001.pdf]

Multimedia Appendix 1

Wearable Device Usability and Acceptability Survey

This appendix presents the survey used to assess user acceptability, usability, and perceived impact of a wrist-worn wearable device in community-dwelling older adults. The survey was originally developed in Chinese and translated into English for reporting.

Section 1. Device Wearing

Q1. How comfortable do you find wearing the wristband (e.g., skin discomfort, strap tightness)?

1 = Very uncomfortable
2 = Uncomfortable
3 = Neutral
4 = Comfortable
5 = Very comfortable

Section 2. Device Usability

Q2. How easy is it to operate the wristband?

1 = Very difficult
2 = Difficult
3 = Neutral
4 = Easy
5 = Very easy

Q3. How clear is the display on the wristband?

1 = Very unclear
2 = Unclear
3 = Neutral
4 = Clear
5 = Very clear

Q4. How satisfied are you with the charging frequency of the wristband?

1 = Very dissatisfied
2 = Dissatisfied
3 = Neutral
4 = Satisfied
5 = Very satisfied

Section 3. App Connectivity and Support

Q5. How often do you experience connectivity or data synchronisation issues (e.g., failure to connect or sync data)?

5 = Never
4 = Rarely (1–2 times per month)
3 = Sometimes (about once per week)
2 = Often (2–3 times per week)
1 = Always (almost daily)

Q6. How often do you need assistance from others when using the device?

5 = Never
4 = Rarely (1–2 times per month)
3 = Sometimes (about once per week)
2 = Often (2–3 times per week)
1 = Always (almost daily)

Section 4. Data Recording and Trust

Q7. How accurate do you think the device is in recording your steps and sleep?

1 = Very inaccurate
2 = Inaccurate
3 = Neutral
4 = Accurate
5 = Very accurate

Q8. To what extent do you trust the data provided by the device?

1 = Do not trust at all
2 = Slightly trust
3 = Neutral
4 = Trust
5 = Trust very much

Section 5. Perceived Lifestyle Impact

Q9. After using the device, do you pay more attention to your daily activities and health?

1 = Not at all
2 = Slightly
3 = Neutral
4 = Yes
5 = Very much

Q10. After using the device, are you more willing to engage in physical activity?

1 = Not at all
2 = Slightly
3 = Neutral
4 = Yes
5 = Very much

Q11. Has your sleep or daily routine changed after using the device?

1 = Much worse
2 = Slightly worse
3 = No change
4 = Slightly improved
5 = Much improved

Section 6. Long-Term Use and Data Sharing

Q12. Are you willing to continue using the device long-term?

1 = Very unwilling
2 = Unwilling
3 = Neutral
4 = Willing
5 = Very willing

Q13. Are you willing to share your device data with your doctor?

1 = Very unwilling
2 = Unwilling
3 = Neutral
4 = Willing
5 = Very willing

Section 7. Open-Ended Questions

Q14. What do you find most helpful about using the device?

Q15. What do you find most inconvenient about using the device?

Q16. Do you have any suggestions for improving the device or its use?
