# Supplementary material for: Real-World Health Monitoring Using Wrist-Worn Wearables in Community-Dwelling Older Adults in China: Mixed Methods Feasibility Study
Source: JMIR Hum Factors. 2026 Oct 29;13:e98592. doi: 10.2196/98592 (PMC13718885; doi:10.2196/98592)
Supplement: Supplementary file 2 [file humanfactors-v13-e98592-s002.pdf]

Multimedia Appendix 3

Description

This appendix presents additional analyses conducted to characterise monitoring patterns, assess sensitivity to alternative monitoring criteria and continuous monitoring measures, examine potential pre-enrolment selection bias, and assess potential survey selection bias.

---

1. Baseline characteristics of enrolled and non-enrolled participants

To assess potential pre-enrolment selection bias, baseline characteristics were compared between the 486 enrolled and 224 non-enrolled participants. No significant differences were observed in age, sex, education, marital status, or self-rated health. Enrolled participants had higher MMSE scores and differed in living arrangement and care arrangement; a statistically significant but small difference was also observed in ADL.

Table 1. Baseline characteristics of enrolled and non-enrolled participants

Characteristic	Enrolled (N=486)	Not enrolled (N=224)	*P* value	Effect size
Age, years: median (IQR)	68 (65-72)	69 (65-72)	.65	ε^2=0.000
Sex, n (%)			.44	Cramér's V=0.029
Male	172 (35.4)	86 (38.4)		
Female	314 (64.6)	138 (61.6)		
Education level, n (%)			.11	Cramér's V=0.079
≤ Junior high school	142 (29.2)	83 (37.1)		
High school/Technical secondary	175 (36)	74 (33)		
≥ College/Undergraduate	169 (34.8)	67 (29.9)		
Marital status, n (%)			.26	Cramér's V=0.042
Married	456 (93.8)	205 (91.5)		
Not currently married	30 (6.2)	19 (8.5)		
Living arrangement, n (%)			<.001	Cramér's V=0.175
With spouse only	334 (68.7)	139 (62.1)		
With children only	51 (10.5)	22 (9.8)		
With spouse and children	37 (7.6)	35 (15.6)		
With relatives/friends	55 (11.3)	15 (6.7)		
Living alone	9 (1.9)	13 (5.8)		
Care arrangement, n (%)			<.001	Cramér's V=0.347
No caregiver	319 (65.6)	66 (29.5)		
Spouse caregiver	150 (30.9)	149 (66.5)		
Child caregiver	15 (3.1)	7 (3.1)		
Relative/friend caregiver	1 (0.2)	2 (0.9)		
Other	1 (0.2)	-		

Self-rated health status, n (%)			.09	Cramér’s V=0.081
Good or very good	125 (25.7)	75 (33.5)		
Fair	323 (66.5)	132 (58.9)		
Poor or very poor	38 (7.8)	17 (7.6)		
MMSE score, median (IQR)	29 (27-30)	26 (24-28)	<.001	ε^2=0.127
Barthel ADL Index, median (IQR)	100 (95-100)	100 (95-100)	.01	ε^2=0.008

Continuous variables were compared using the Kruskal-Wallis test, with epsilon-squared (ε^2) reported as the effect size. Categorical variables were compared using Pearson’s chi-square test, with Cramér’s V reported as the effect size; a Monte Carlo procedure was used for care arrangement because of sparse expected cell counts. MMSE=Mini-Mental State Examination; ADL=activities of daily living; IQR=interquartile range.

2. Characterisation of participants with limited valid monitoring

To characterise participants with no valid monitoring day, continuous heart-rate records were used to examine observed wear time among the 106 participants in this group. All 106 participants had at least some heart-rate data, but observed wear was very limited. Most participants (97/106, 91.5%) had a maximum daily wear time of <1 hour, while the remaining 9 (8.5%) had a maximum daily wear time between 1 and <5 hours. These findings indicate that the no-valid-monitoring-day category predominantly reflected very limited wear rather than substantial wear that narrowly failed to meet the ≥10-hour valid-day criterion.

Table 2. Maximum observed daily wear time among participants with no valid monitoring day

Maximum observed daily wear time	n	%
<1 hour	97	91.5
1 to <5 hours	9	8.5
Total	106	100

To characterise heterogeneity within the non-consecutive valid monitoring category, the distribution of valid monitoring days was examined among the 156 participants in this group. Most participants had only one or two valid monitoring days (148/156, 94.9%), while 8 (5.1%) had ≥3 valid days that never occurred on three consecutive days.

Table 3. Heterogeneity within the non-consecutive valid monitoring category

Monitoring pattern	n	% of non-consecutive group (n=156)
1 valid monitoring day	119	76.3
2 valid monitoring days	29	18.6
≥3 valid days, but never ≥3 consecutively	8	5.1

3. Sensitivity to alternative consecutive valid monitoring criteria

To assess the robustness of findings to the primary ≥3-consecutive-valid-day criterion, progressively stricter thresholds (≥4, ≥5, and ≥7 consecutive valid days) and continuous

measures of monitoring extent and persistence were examined. As the threshold increased, the proportion meeting the criterion decreased from 58.9% at ≥3 days to 46.8% at ≥7 days.

Table 4. Sensitivity to alternative consecutive valid monitoring criteria

**Consecutive-valid-day criterion**	**Meeting criterion, n**	**% of participants with ≥1 valid day (n=380)**
≥3 days	224	58.9
≥4 days	211	55.5
≥5 days	196	51.6
≥7 days	178	46.8

Sensitivity analyses were restricted to the 380 participants with ≥1 valid monitoring day because the purpose was to examine how classification changed across alternative consecutive-valid-day thresholds among participants with at least one valid monitoring day. Participants with no valid monitoring day (n=106) were therefore excluded from these analyses.

To further characterise monitoring extent and persistence beyond the categorical criterion, total valid monitoring days and the maximum consecutive-valid-day streak were examined among the 224 participants with minimal consecutive valid monitoring. Considerable variation was observed in both measures.

Table 5. Continuous monitoring measures among participants with minimal consecutive valid monitoring

**Measure**	**n**	**Median**	**IQR**	**Range**
Total valid monitoring days	224	60.5	19-94.3	3-409
Maximum consecutive-valid-day streak, days	224	16	8-34	3-168

To assess whether the observed association with self-rated health was sensitive to the choice of monitoring criterion, the analysis was repeated using the ≥3-, ≥4-, ≥5-, and ≥7-consecutive-valid-day thresholds. Poorer self-rated health was consistently associated with lower odds of meeting the monitoring criterion across all four thresholds.

Table 6. Sensitivity of the self-rated health association to alternative thresholds

**Criterion**	**Meeting criterion, n**	**OR per one-category worsening in self-rated health**	**95% CI**	***P* value**
≥3 consecutive days	224	0.67	0.48-0.92	.02
≥4 consecutive days	211	0.61	0.43-0.83	.002
≥5 consecutive days	196	0.66	0.48-0.90	.01
≥7 consecutive days	178	0.69	0.50-0.93	.02

Odds ratios (ORs) are from unadjusted logistic sensitivity analyses. Higher self-rated-health scores indicate poorer perceived health; therefore, ORs <1 indicate lower odds of meeting the criterion with poorer self-rated health.

4. User experience survey according to wearable monitoring pattern

To assess potential survey selection bias, survey participation was examined across wearable monitoring patterns. Overall, 106/486 participants (21.8%) completed the user experience survey. Response rates were highest among participants with minimal consecutive valid monitoring (74/224, 33%), compared with those with non-consecutive valid monitoring (21/156, 13.5%) or no valid monitoring day (11/106, 10.4%).

Table 7. Survey participation according to wearable monitoring pattern

Monitoring category	Total participants	Survey respondents	Response rate
Minimal consecutive valid monitoring	224	74	33%
Non-consecutive valid monitoring	156	21	13.5%
No valid monitoring day	106	11	10.4%
Total	486	106	21.8%

To examine whether survey respondents differed from nonrespondents in measured baseline characteristics, demographic, cognitive, and functional characteristics were compared between the two groups. Respondents and nonrespondents were broadly comparable, although respondents had slightly higher MMSE scores (*P*=.046), with a very small effect size. No other measured baseline characteristics differed significantly.

Table 8. Baseline characteristics of survey respondents and nonrespondents

**Characteristic**	**Respondents (n=106)**	**Nonrespondents (n=380)**	***P* value**
Age, years, median (IQR)	67.5 (65-71)	68.0 (65-72)	.15
Sex, n (%)			.09
Male	30 (28.3)	142 (37.4)	
Female	76 (71.7)	238 (62.6)	
Education level, n (%)			.11
≤ Junior high school	23 (21.7)	119 (31.3)	
High school/technical secondary	39 (36.8)	136 (35.8)	
≥ College/undergraduate	44 (41.5)	125 (32.9)	
Marital status, n (%)			>.99
Married	100 (94.3)	356 (93.7)	
Not currently married	6 (5.7)	24 (6.3)	
Living arrangement, n (%)			.85
With spouse only	70 (66)	264 (69.5)	
With children only	12 (11.3)	39 (10.3)	
With spouse and children	9 (8.5)	28 (7.4)	
With relatives/friends	14 (13.2)	41 (10.8)	
Living alone	1 (0.9)	8 (2.1)	
Self-rated health, n (%)			.35
Good or very good	33 (31.1)	92 (24.2)	

Fair	65 (61.3)	258 (67.9)	
Poor or very poor	8 (7.5)	30 (7.9)	
MMSE score, median (IQR)	29.0 (28-30)	29.0 (27-30)	.046
ADL score, median (IQR)	100.0 (100-100)	100.0 (95-100)	.13

Data are presented as n (%) or median (IQR). Continuous variables were compared using the Kruskal–Wallis test and categorical variables using Pearson's chi-square test or exact procedures where appropriate. MMSE=Mini-Mental State Examination; ADL=activities of daily living; IQR=interquartile range.

To further assess survey selection bias, wearable monitoring patterns and monitoring persistence were compared between survey respondents and nonrespondents. Respondents were more likely to have minimal consecutive valid monitoring (69.8% vs 39.5%) and, among participants with minimal consecutive valid monitoring, had more total valid monitoring days and longer maximum consecutive-valid-day streaks than nonrespondents.

Table 9. Wearable monitoring characteristics of survey respondents and nonrespondents

**Monitoring measure**	**Respondents (n=106)**	**Nonrespondents (n=380)**	***P* value**
Monitoring category, n (%)			<.001
Minimal consecutive valid monitoring	74 (69.8%)	150 (39.5%)	
Non-consecutive valid monitoring	21 (19.8%)	135 (35.5%)	
No valid monitoring day	11 (10.4%)	95 (25%)	
Monitoring persistence among participants with minimal consecutive valid monitoring			
Total valid monitoring days, median (IQR)	82 (47-121)	40 (14-86.5)	<.001
Maximum consecutive-valid-day streak, median (IQR)	22 (11-50)	13 (7-30)	.002
