# Supplementary material for: Real-World Health Monitoring Using Wrist-Worn Wearables in Community-Dwelling Older Adults in China: Mixed Methods Feasibility Study
Source: JMIR Hum Factors. 2026 Oct 29;13:e98592. doi: 10.2196/98592 (PMC13718885; doi:10.2196/98592)
Supplement: Supplementary file 3 [file humanfactors-v13-e98592-s003.pdf]

Multimedia Appendix 2

Description

This appendix presents quantitative and qualitative results from the wearable device usability and acceptability survey, including Likert-scale ratings and thematic analysis of open-ended responses.

Table 1. Summary of Likert-scale ratings (1–5) for usability and acceptability items (N=106)

Question	Item	Mean	SD	Median	IQR
Q1	Device comfort	3.87	1.00	4	3-5
Q2	Ease of use	3.65	1.07	4	3-4
Q3	Display readability	3.81	0.76	4	4-4
Q4	Charging frequency	3.80	0.81	4	3.25-4
Q5	Connectivity issues*	3.58	1.09	4	3-4
Q6	Need for assistance*	4.22	0.96	4	4-5
Q7	Recording accuracy	3.51	0.88	4	3-4
Q8	Trust in data	3.79	0.71	4	3-4
Q9	Health awareness	3.95	0.61	4	4-4
Q10	Increased physical activity	3.84	0.72	4	3-4
Q11	Changes in daily habits	3.53	0.66	3	3-4
Q12	Willingness for long-term use	3.95	0.70	4	4-4
Q13	Willingness to share data	4.05	0.59	4	4-4

* Higher scores indicate fewer issues (Q5) or less need for assistance (Q6).

Table 2. Perceived benefits of wearable device use (open-ended responses)

Theme	n (%)
Sleep monitoring	38 (35.8)
Activity/exercise monitoring	37 (34.9)
Heart rate/SpO_2 monitoring	29 (27.4)
Health management awareness	29 (27.4)
Routine and schedule reminders	7 (6.6)
Other daily support functions	3 (2.8)
No specific benefits	4 (3.8)

Table 3. Reported barriers and inconveniences

Theme	n (%)
No issues	36 (34)
Device discomfort	18 (17)
Data synchronisation issues	12 (11.3)
Short battery life	12 (11.3)
Difficult to use	9 (8.5)
Difficulty forming habit	7 (6.6)
Unclear display/layout	6 (5.7)
Limited features	5 (4.7)

Table 4. Suggestions for improvement

Theme	n (%)
Additional health monitoring (e.g., blood pressure, glucose)	35 (33)
No suggestions / satisfied	27 (25.5)
Simplify operation / guidance	17 (16)
Improve comfort or battery life	12 (11.3)
Improve display	10 (9.4)
Improve data accuracy or upload	9 (8.5)
More personalisation	1 (0.9)
